# Peripheral and Central Neuroinflammatory Changes and Pain Behaviors in an Animal Model of Multiple Sclerosis

**DOI:** 10.3389/fimmu.2016.00369

**Published:** 2016-09-22

**Authors:** Samuel S. Duffy, Chamini J. Perera, Preet G. S. Makker, Justin G. Lees, Pascal Carrive, Gila Moalem-Taylor

**Affiliations:** ^1^School of Medical Sciences, UNSW Medicine, The University of New South Wales (UNSW Australia), Sydney, NSW, Australia

**Keywords:** multiple sclerosis, experimental autoimmune encephalomyelitis, pain, neuroinflammation, glia, T cells

## Abstract

Pain is a widespread and debilitating symptom of multiple sclerosis (MS), a chronic inflammatory demyelinating disease of the central nervous system. Although central neuroinflammation and demyelination have been implicated in MS-related pain, the contribution of peripheral and central mechanisms during different phases of the disease remains unclear. In this study, we used the animal model experimental autoimmune encephalomyelitis (EAE) to examine both stimulus-evoked and spontaneous pain behaviors, and neuroinflammatory changes, over the course of chronic disease. We found that mechanical allodynia of the hind paw preceded the onset of clinical EAE but was unmeasurable at clinical peak. This mechanical hypersensitivity coincided with increased microglial activation confined to the dorsal horn of the spinal cord. The development of facial mechanical allodynia also emerged in preclinical EAE, persisted at the clinical peak, and corresponded with pathology of the peripheral trigeminal afferent pathway. This included T cell infiltration, which arose prior to overt central lesion formation and specific damage to myelinated neurons during the clinical peak. Measurement of spontaneous pain using the mouse grimace scale, a facial expression-based coding system, showed increased facial grimacing in mice with EAE during clinical disease. This was associated with multiple peripheral and central neuroinflammatory changes including a decrease in myelinating oligodendrocytes, increased T cell infiltration, and macrophage/microglia and astrocyte activation. Overall, these findings suggest that different pathological mechanisms may underlie stimulus-evoked and spontaneous pain in EAE, and that these behaviors predominate in unique stages of the disease.

## Introduction

Multiple sclerosis (MS) is a chronic disease of the central nervous system (CNS) characterized by widespread focal areas of inflammation, demyelination, gliosis, and neurodegeneration. Typical onset is between the ages of 20 and 30, and it is the most common cause of chronic neurological disability in early to middle adult life (1–3). Of the multitude of sensory, cognitive, and motor symptoms associated with the disease, pain has recently been estimated to have a lifetime prevalence of 66.5% (4). It severely impacts the quality of life of sufferers and is particularly difficult to treat (5, 6). Pain in MS may be nociceptive (arising as a result of non-neural tissue damage by activation of nociceptors), neuropathic (a consequence of direct damage to the somatosensory system), or mixed. Specific conditions include trigeminal neuralgia and Lhermitte’s phenomenon (neuropathic; due to ectopic impulse generation along primary afferents), ongoing extremity pain (neuropathic; secondary to lesion formation in the spino–thalamo–cortical pathways), painful tonic spasms and spasticity pain (mixed; mediated by nociceptors and arises secondary to lesions in the central motor pathways), pain associated with optic neuritis (nociceptive; originating from *nervi nervorum*), musculoskeletal pains (nociceptive; secondary to motor disorders), and migraine (nociceptive; resulting from predisposition or secondary to midbrain lesions) (7). As it stands, there is a dire need for effective and targeted therapies aimed at the amelioration of pain in MS. This is an issue that, at least in part, stems from a lack of reliable and translatable pain outcome measures in animal models of MS.

Experimental autoimmune encephalomyelitis (EAE) is the most commonly used experimental animal model of MS, which shares many key pathological characteristics with the human disease; namely neuroinflammation, demyelination, and neuronal damage (8). Further, mice with EAE have been shown to develop pain behaviors including thermal hyperalgesia and mechanical and cold allodynia of the tail, hind paws, and fore paws in mild and preclinical EAE (9–14). An obvious limitation of these approaches arises from the confounding tail and hind limb motor impairment characteristic of clinical EAE, which impedes the testing of pain behaviors due to absent withdrawal reflexes during periods of paralysis. A recent study demonstrated that mice with EAE develop increased sensitivity to air puffs applied to the whisker pad indicating altered facial sensitivity in EAE (15). Although unclear, it is widely believed that stimulus-evoked pain behaviors in EAE occur as a result of inflammation, glial activation, and demyelination in areas involved in the processing of painful stimuli such as the dorsal horn of the spinal cord (SC) (10) and trigeminal afferent pathways (15). Most recently, the early emergence of neuropathic pain-like behaviors in the sequelae of EAE has been investigated and was shown to be associated with altered excitatory–inhibitory balance within the primary somatosensory cortex (16) and increased expression of the neuronal injury marker activating transcription factor-3 (ATF-3) in sensory neurons of the peripheral ganglia (17), both of which arose prior to an overt adaptive immune response and motor symptoms.

The study of pain in EAE has so far been focused on measures of hyperesthesia, which while nonetheless useful, may prove problematic when attempting to translate analgesic therapies optimized in mice to the treatment of spontaneous forms of neuropathic pain in MS patients. We hypothesized that mice with EAE develop spontaneous pain in addition to stimulus-evoked pain and aimed to examine such pain behaviors in association with neuroinflammatory changes over the course of chronic EAE. We show, for the first time, that in addition to stimulus-evoked pain (facial allodynia measured by whisker pad sensitivity to mechanical stimuli) mice with EAE also develop spontaneous pain (facial grimacing measured by the mouse grimace scale, a facial expression-based pain coding system), an important component of MS-associated pain. Comprehensive analysis of immune, glial, and neural changes in correspondence with observable pain behaviors shows that distinct stages of EAE are associated with specific pain phenotypes and neuroinflammation in both the peripheral and CNS.

## Materials and Methods

### Animals

Female C57BL/6J mice aged 10–12 weeks (Australian Biological Resources, Moss Vale, NSW, Australia) were used in all experiments. Mice were housed in individually ventilated cages with water and food *ad libitum* in groups of 3–5 and maintained on a 12-h light–dark cycle. The facility was kept at a constant room temperature (RT) and humidity, and the animals were monitored daily throughout experiments. All experiments were approved by the Animal Care and Ethics Committee of the University of New South Wales, Sydney, NSW, Australia.

### EAE Induction and Assessment

Experimental autoimmune encephalomyelitis was induced by subcutaneous immunization with myelin oligodendrocyte glycoprotein 35–55 (MOG_35–55_) emulsified in complete Freund’s adjuvant (CFA). Emulsions were purchased from Hooke Laboratories (Lawrence, MA, USA) as prefilled syringes, each containing ~1 mg/mL MOG_35–55_ emulsified with 2–5 mg killed *Mycobacterium tuberculosis* H37Ra/mL in incomplete Freund’s adjuvant. Control mice were immunized with CFA alone (Hooke Laboratories; at the same concentration given to mice immunized with MOG_35–55_/CFA). Immunizations were given under 3–5% isoflurane anesthesia in oxygen as 2 × 100 μL subcutaneous injections; one at the base of the tail and one on the upper back. An intraperitoneal injection of 200 ng Pertussis Toxin (Hooke Laboratories) in 100 μL Dulbecco’s Phosphate Buffered Saline (D-PBS; Life Technologies Pty Ltd., VIC, Australia) was given to all mice 2–6 h after subcutaneous immunization, and again 22–26 h later.

Post-induction, mice were monitored daily for body weight and EAE clinical scores according to a detailed EAE grading system supplied by Hooke Laboratories. Briefly, EAE clinical scores were assigned as follows: Grade 1 = limp tail; Grade 2 = limp tail and weakness of hind legs; Grade 3 = limp tail and complete paralysis of hind legs or limp tail with paralysis of one front and one hind leg; Grade 4 = limp tail and complete hind leg and partial front leg paralysis; Grade 5 = complete hind and complete front leg paralysis.

### Measurement of Hind Paw Mechanical Allodynia

Hind paw mechanical allodynia was assessed by placing mice in 10 × 10 cm red-tinted chambers on an elevated wire mesh. Mice were habituated to the behavioral testing apparatus twice for 1 h each prior to baseline testing. Mechanical allodynia of the hind paws was assessed using a set of calibrated von Frey filaments. These were applied to the mid-plantar surface of the hind paw until bending and maintained for 3 s. Paw withdrawals were noted as swift, sharp responses to application of the filament, and testing was conducted blind to experimental groups using Dixon’s up-and-down method (18). A 50% paw withdrawal threshold (PWT) per animal was found by averaging the 50% PWT calculated for each paw.

### Measurement of Facial Mechanical Allodynia

Facial allodynia was assessed using a method previously described by Lyons et al. to measure facial pain in a model of trigeminal inflammatory compression injury (19). In the week prior to baseline behavioral testing, animals were handled daily using a cotton glove in order to gradually acclimatize the mice to being gently restrained in the experimenter’s hand. Prior to testing, the same experimenter gently restrained the mouse in their palm with the head exposed using the cotton glove until the mouse was acclimated and calm. During testing, a second experimenter blinded to experimental groups applied a 0.07 g von Frey filament to the whisker pad five times per side, with a 1-min interval between tests. Responses were recorded as head withdrawal, fore paw swiping, or facial flinching by a blinded experimenter, and a percentage of total responses over the five tests per side were calculated for each animal.

### Mouse Grimace Scale

Mice were habituated for 15 min once prior to baseline testing in a 5 × 5 × 10 cm plastic arch with glass windows at each end placed on an elevated wire mesh. During testing, mice were placed in the same arch, with two Canon 500D cameras positioned at each end for high definition video recording. Mice were filmed for 11 min total, and screen grabs were later taken at each minute mark following beginning of recording for a total of 10 photos per mouse. These were taken as soon as a clear head shot could be observed and scored according to the criteria developed by Langford and colleagues (20) with the omission of the whisker change action unit as this was deemed difficult in C57BL/6J mice due to coat color (Figure 2A). Mean mouse grimace scale (MGS) difference scores from baseline were calculated in a blinded manner at time points tested post-EAE induction.

### Flow Cytometry

At designated end-points post-EAE induction (day 8 pre-onset, day 16 clinical peak, and day 32 chronic phase), mice were euthanized using 0.1 mL of Lethabarb (Virbac, NSW, Australia), injected i.p. and were transcardially perfused with heparinised 0.9% saline solution. The L3–5 dorsal root ganglia (DRG; left and right), SC, brain, and trigeminal tissue (nerve and ganglia; right) were then dissected and placed into PBS on ice. Tissues were coarsely chopped and incubated with 1 mL of Accutase (Sigma Aldrich, NSW, Australia) for 30 min at 37°C and 5% CO_2_. Tissues were then mechanically ground through 70 μm cell strainers (BD Biosciences, Franklins Lakes, NJ, USA) in 10 mL of PBS. Cell suspensions were centrifuged for 5 min at 1000 × *g* at 4°C, before discarding the supernatant. 10 mL of 30% Percoll (GE Healthcare Australia Pty Ltd., NSW, Australia) in PBS was added to each sample, which were then centrifuged for 25 min at 600 × *g* at RT. The supernatant, including the myelin/cell debris layer, was carefully removed using a pipette, and the cell pellet was resuspended in 1 mL of autoMACS (magnetic-activated cell sorting; Miltenyi Biotec Australia Pty Ltd., NSW, Australia) running buffer. Samples were centrifuged for 5 min at 600 × *g* at 4°C, before removing as much of the supernatant from the pellet as possible using a pipette. Cells were counted and PBS added to give a final cell concentration of ~1 × 10^7^ cells per mL.

One hundred microliters of cells from each sample were divided into separate tubes, and 1 μL of Zombie Violet cell viability dye (Biolegend, CA, USA) was added to each tube, which were incubated in the dark for 15 min at RT. Cells were washed once in 1 mL of autoMACS running buffer by centrifuging for 5 min at 600 × *g* at 4°C, the supernatant was discarded and the residual volume was incubated with 1 μL anti-mouse CD16/CD32 Fc Block (eBioscience, CA, USA) for 5 min at RT. For analysis of premyelinating and myelinating oligodendrocytes, primary anti-galactocerebroside (GALC) and anti-MOG antibodies (Merck Millipore, VIC, Australia) were first conjugated to unique fluorophores using Lightning-Link Antibody Labeling kits (Novus Biologicals, Littleton, CO, USA) as per manufacturer’s instructions. Anti-GALC-PE and anti-MOG-FITC conjugated antibodies were then incorporated into a standard flow cytometry staining protocol as follows. Antibodies including anti-CD45-APC (eBioscience; 1:1000), anti-CD4-FITC (eBioscience; 1:1000), Anti-GALC-PE (1:200), and anti-MOG-FITC (1:200) diluted in 100 μL of autoMACS running buffer were added to each sample, and cells were incubated for 30 min at 4°C in the dark. Appropriate isotype and fluorescence minus one controls were included by staining 100 μL of pooled samples for each tissue analyzed. After staining, cells were washed three times in 1 mL of autoMACS running buffer before being resuspended in 0.2 mL of the same buffer for analysis on a BD FACS Canto II flow cytometer. A minimum of 50,000 events was acquired per sample, and data were analyzed using FlowJo software (FlowJo, OR, USA).

### Immunohistochemistry

At designated end-points post-EAE induction (day 8 pre-onset and day 16 clinical peak), mice were euthanized and transcardially perfused with heparinised 0.09% saline solution followed by 10% formalin solution (Sigma Aldrich). The L3–5 DRG, L3–5 SC, brain (including brain stem), and trigeminal tissue was then dissected and post-fixed in formalin solution overnight at 4°C. The medulla was then dissected from the brain, and all tissues were transferred to 30% sucrose + 0.1% sodium azide solution and stored at 4°C until sectioning. Sections were cut using a cryostat (Leica Biosystems, Buffalo grove, IL, USA), with DRGs, and trigeminal tissue samples sectioned longitudinally at a thickness of 10 μm, and SC and medulla samples sectioned coronally at a thickness of 20 μm. All sections were cut sequentially such that each slide contained 4–6 serial sections representative of the entire thickness of the tissue. Sections were transferred directly to gelatin-coated glass slides, air dried overnight, and stored at −80^o^C until staining.

Prior to staining, sections were fixed with ethanol for 10 min at RT. Sections were then washed twice with distilled water and once with PBS containing 0.05% Tween-20 (PBS-T). A blocking solution containing PBS with 5% donkey or goat serum, 0.2% Tween-20, and 0.3% Triton X-100 was applied to each slide for incubation at RT for 1 h in the dark. The blocking solution was drained, and sections were incubated with the following antibodies diluted in PBS containing 5% bovine serum albumin and 0.03% Triton-X: rat anti-mouse CD3 (T cells; 1:100; R&D systems, MN, USA), rabbit anti-mouse/rat IBA-1 (ionized calcium-binding adaptor-1; macrophages/microglia; 1:2000; Wako Chemicals USA, Richmond, VA, USA), mouse anti-mouse GFAP (glial fibrillary acidic protein; activated astrocytes; 1:2000; Chemicon, Temecula, CA, USA), rabbit anti-mouse ATF-3 (activating transcription factor-3; cell damage; 1:400; Santa Cruz Biotechnology Inc., TX, USA), goat anti-mouse CGRP (calcitonin gene-related peptide; peptidergic neurons; 1:1000; Abcam, VIC, Australia), anti-mouse IB4-FITC (isolectin B4; non-peptidergic neurons; 1:100; Sigma Aldrich), and rabbit anti-mouse NF200 (neurofilament 200; myelinated neurons; 1:1000; Sigma Aldrich). IBA-1 and GFAP primary antibody incubation was conducted at RT for 1 h, while all other primary antibodies were incubated overnight at 4°C. Sections were then washed four times with PBS-T for 10 min each, before adding secondary antibodies, which included: Alexa Fluor 488 conjugated donkey anti-mouse (1:1000; Life Technologies, Mulgrave, VIC, Australia), Alexa Fluor 546 conjugated donkey anti-rabbit (1:1000; Life Technologies), or Cy3 conjugated donkey anti-rabbit (1:1000; Jackson ImmunoResearch Laboratories Inc., PA, USA) in the same buffer as the primary antibody. Negative staining controls were incorporated whereby tissues were incubated with secondary antibody alone in the absence of the relevant primary antibody (to control for non-specific binding of the secondary antibody). Following a 1 h incubation at RT in the dark, sections were washed four times in PBS-T for 10 min each. Prolong gold anti-fade reagent with 4′, 6-diamidino-2-phenylindole (DAPI; Life Technologies) was applied before slides were cover slipped and stored at 4°C until viewing.

### Image Analysis

Analysis of images was conducted blind to experimental groups from which the tissue was derived.

#### Trigeminal Ganglia and Dorsal Root Ganglia

For each animal, slides containing 4–6 sections of the L3–5 DRGs (left and right, with two of each L3–5 DRG represented in each section) or trigeminal tissue (left) were stained for a given antibody. For DRGs, images were taken of 2–3 DRGs contained in each section (a total of 12–18 images per slide/animal). In trigeminal ganglia (TG), images of areas containing cell bodies (inclusive of the ophthalmic, maxillary, and mandibular distributions of ganglia) were taken at 20× magnification for each section (a total of 12–18 images per slide/animal). Using Image J software, immunoreactive cells were either manually counted and expressed per area of ganglion (CD3 analyzes), counted and expressed as a percentage of total cell bodies in the ganglia of interest (ATF-3 and neuropeptide analyses), or expressed as a percentage of immunoreactivity after subtracting background (IBA-1 analyses). Values for each image of ganglia regions were averaged to give a mean immunoreactivity per animal.

#### Trigeminal Nerve and Trigeminal Root Entry Zone

Slides containing 4–6 sections of left trigeminal tissue per animal were stained for a given antibody. Images were taken at 20× magnification of regions immediately distal to the CNS-peripheral nervous system (PNS) junction (trigeminal nerve; TN images) and just proximal to the CNS–PNS junction at the trigeminal root entry zone (TREZ) (4–6 images per slide/animal). Immunoreactive cells were either manually counted and expressed per area of nerve (CD3 analyses) or expressed as a percentage of immunoreactivity after subtracting background (IBA-1 analyses) using Image J software. Values for each section were averaged to give the mean immunoreactivity per animal.

#### Spinal Cord and Spinal Trigeminal Nucleus

Slides containing 4–6 sections of L3–5 SC or medulla per animal were stained for a given antibody. For SC sections, images were taken at 20× magnification of the left and right ventral and dorsal horn regions (8–12 images of ventral horn, and 8–12 images of dorsal horn regions per animal/slide). Sections of the medulla were taken around the level of the obex in the caudal medulla in order to visualize the region corresponding to the spinal trigeminal nucleus (STN) (sections were inclusive of the subnucleus caudalis, subnucleus interpolaris, and subnucleus oralis). Images were taken at 20× magnification in regions of the left and right STN (8–12 images per animal/slide). Image J software was used to calculate the percentage of total immunoreactivity after subtracting background. Values for each region were averaged across sections to give the mean immunoreactivity per animal.

#### Image Acquisition

All images were viewed using Olympus BX51 epifluorescence microscope, and images captured using an Olympus DP73 camera using cellSens software (Olympus, Tokyo, Japan). All images for a particular stain were taken using identical microscope settings.

### Statistical Analysis

All statistical analyses were conducted using GraphPad Prism 6 software. Since pain behavioral data sets involving the testing of mechanical allodynia proved to be not normally distributed using a Shapiro–Wilk normality test, a non-parametric Mann–Whitney test was used as the most appropriate statistical test for comparing two groups – EAE mice vs. control mice at each time point. The Holm–Bonferroni correction for multiple comparisons was then used to account for multiple testing. MGS data were analyzed using a repeated measures two-way ANOVA (treatment; time) with Tukey’s *post hoc* test. Immunohistochemistry data were analyzed using an unpaired student’s *t*-test (when comparing two groups; EAE mice vs. control mice) or a one-way ANOVA with Tukey’s *post hoc* test (when comparing greater than two groups in neuropeptide analyses) at individual time points. Multiple time point flow cytometry data were analyzed using two-way ANOVA (treatment; time) with Tukey’s *post hoc* test. Pain behavioral data sets analyzed using non-parametric statistical tests (hind paw and facial allodynia) are presented as box and whisker plots, where box limits show the first and third quartile, the center line is the median and the whiskers represent the minimum and maximum values. MGS, flow cytometry, and immunohistochemistry data are presented as arithmetic mean ± SEM. The level of significance was set as *p* < 0.05 for all analyses.

## Results

### Increased Stimulus-Evoked Pain and Facial Grimacing in Mice with EAE

**Figure 1:**
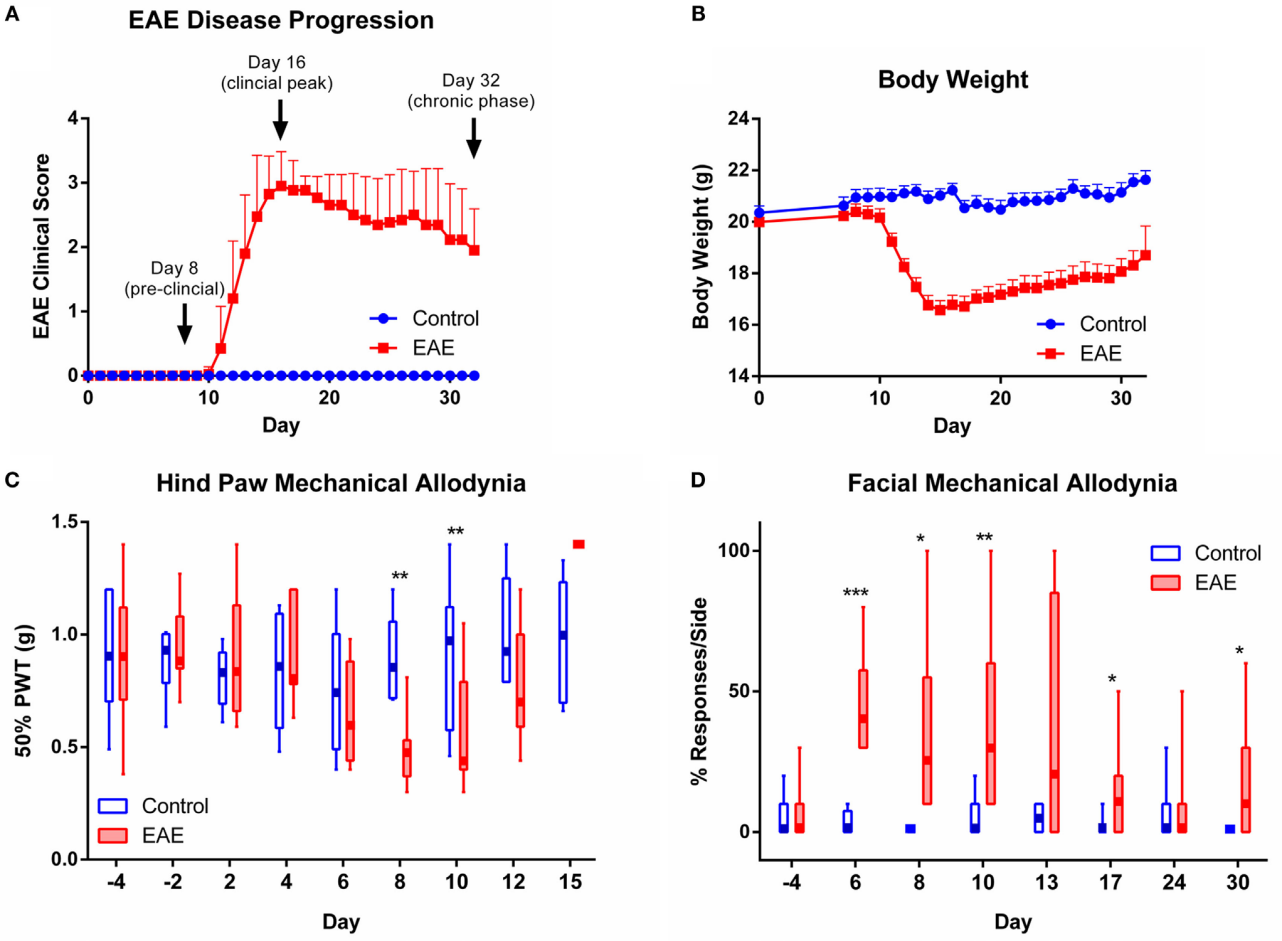
**EAE disease progression and evoked pain behaviors observed over the course of chronic EAE**. Disease progression of EAE **(A)** and **(B)** body weight over the 32-day monitoring period post-induction in control and EAE mice (*n* = 20). Time points chosen for analysis of nervous tissue at preclinical, peak, and chronic phases of disease are indicated in **(A)**. **(C)** Mechanical allodynia of the hind paw observed in the preclinical period in mice with EAE. At days 8 and 10 post-induction, EAE mice showed significantly reduced 50% paw withdrawal thresholds (PWTs) compared to control mice (*n* = 6–15). **(D)** Facial allodynia observed in mice over the course of chronic EAE. At days 6, 8, 10, 17 and 30 post-induction, EAE mice recorded a significantly increased percentage of responses to the mechanical stimulus applied to the whisker pad compared to control mice (*n* = 4–9). *P < 0.05, **P < 0.01, and ***P < 0.001, Mann–Whitney test with Holm–Bonferroni correction for multiple comparisons. EAE clinical scores and body weight data are expressed as mean ± SEM, while pain behavioral data are expressed as box and whisker plots where box limits show the first and third quartile, the center line is the median, and the whiskers represent the minimum and maximum values.

To investigate pain symptoms during the course of chronic EAE, mice were immunized with the MOG_35–55_ peptide in CFA. Mice developed chronic EAE with typical disease onset between 10 and 13 days post-induction, a mean peak clinical score of 3 on day 16, and a gradual, partial recovery to a mean clinical score of 2 by day 32 post-induction. Control mice immunized with CFA alone did not develop any clinical signs of EAE over the 32-day monitoring period (Figure 1A). Clinical EAE was accompanied by a significant loss of body weight, which was most severe at time points corresponding to the EAE clinical peak (Figure 1B).

To assess stimulus-evoked pain, we first tested mechanical pain hypersensitivity in the hind paws (Figure 1C). Compared to controls, EAE mice showed reduced 50% PWTs during the pre-clinical stage of disease on days 8 and 10 post-induction (*n* = 6–15, *p* < 0.01). Corresponding to the onset of clinical EAE and accompanying hind limb motor impairment, the 50% PWTs of EAE mice showed a sharp rise on day 15 (clinical peak of disease) where PWTs rose above baseline levels and reached the imposed upper limit of von Frey testing in all animals (1.4 g; Figure 1C). Testing of mechanical pain hypersensitivity in the hind paws was terminated at disease peak due to paralysis. The restrictions imposed by hind limb motor confounds on continual testing of mechanical allodynia in clinical EAE prompted us to use a technique developed for testing mechanical pain in the face (19), which is not affected by motor deficits. We found that EAE mice were significantly more responsive to mechanical stimuli applied to the whisker pad than control mice over the entire course of chronic EAE, as seen on days 6, 8, 10, 17, and 30 (Figure 1D, *n* = 4–9, *p* < 0.05–0.001).

**Figure 2:**
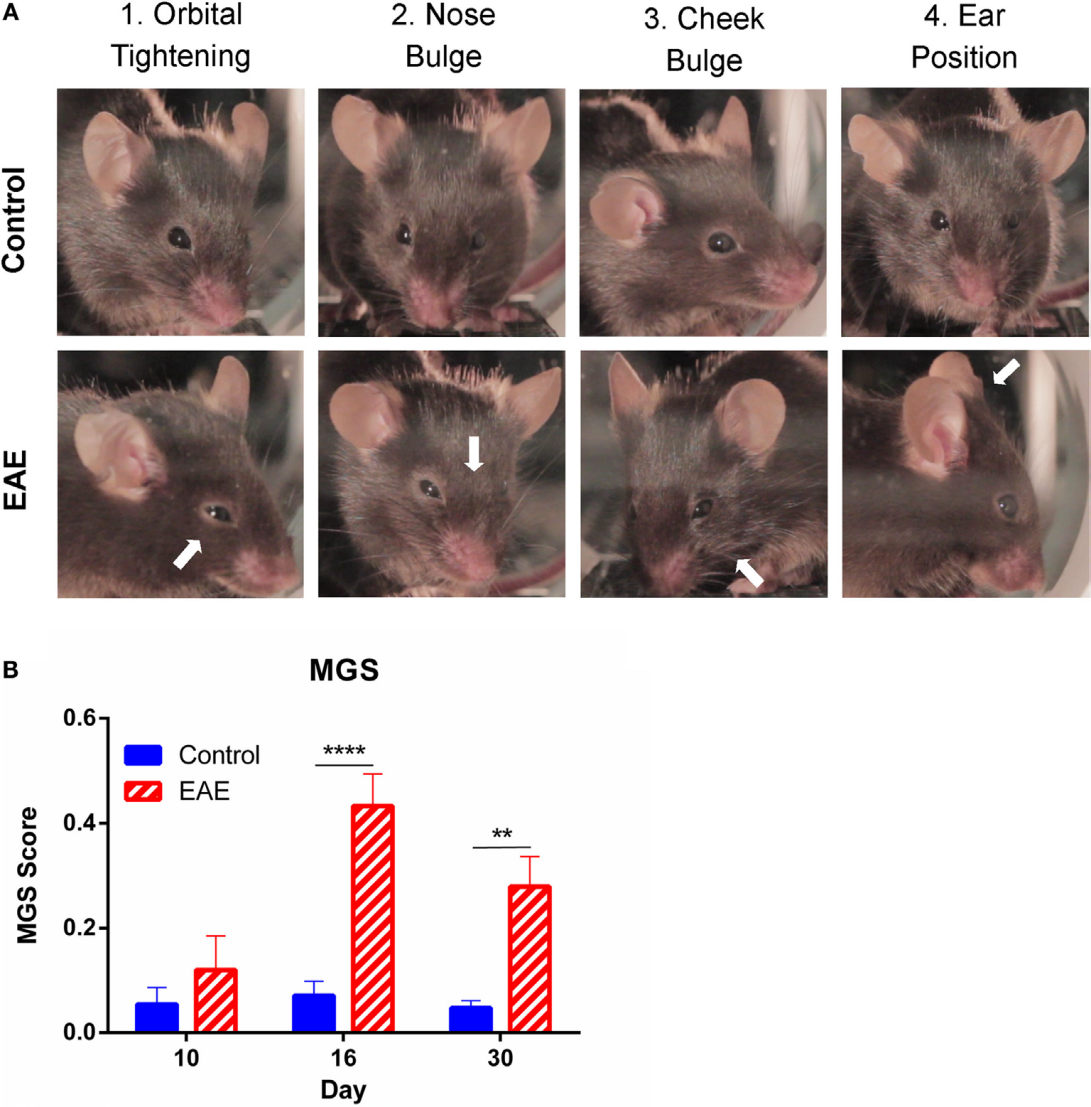
**Facial grimacing observed over the course of chronic EAE**. **(A)** Representative images of action units analyzed as part of the mouse grimace scale (MGS) scoring. Control images depict an MGS score of 0 for each of the given action units, while white arrows indicate action units which scored 1–2 on the MGS in EAE mice. **(B)** Mean MGS difference scores calculated from control and EAE mice over the course of chronic EAE. Significant increases in MGS scores were seen at days 16 and 30 in EAE mice compared to control mice (*n* = 7–8). **P < 0.01 and ****P < 0.0001, repeated measures two-way ANOVA followed by Tukey’s *post hoc* test. Data are expressed as mean ± SEM.

To assess spontaneous (as opposed to experimenter-evoked) pain in EAE, we utilized the MGS (Figure 2A), a standardized murine facial expression-based coding system (20). This allowed both continuous testing of pain behaviors during paralytic clinical disease, and the assessment of spontaneous pain in EAE, which has not yet been explored in the model. No change in mean MGS scores were seen in the preclinical period (day 8); however, the mean MGS scores in EAE mice were higher at day 16 (*n* = 7–8, *p* < 0.01) and day 30 (*n* = 7–8, *p* < 0.01) post-EAE induction as compared to control mice (Figure 2B).

### Changes in the Proportions of Premyelinating and Myelinating Oligodendrocytes in the CNS of Mice with EAE

**Figure 3:**
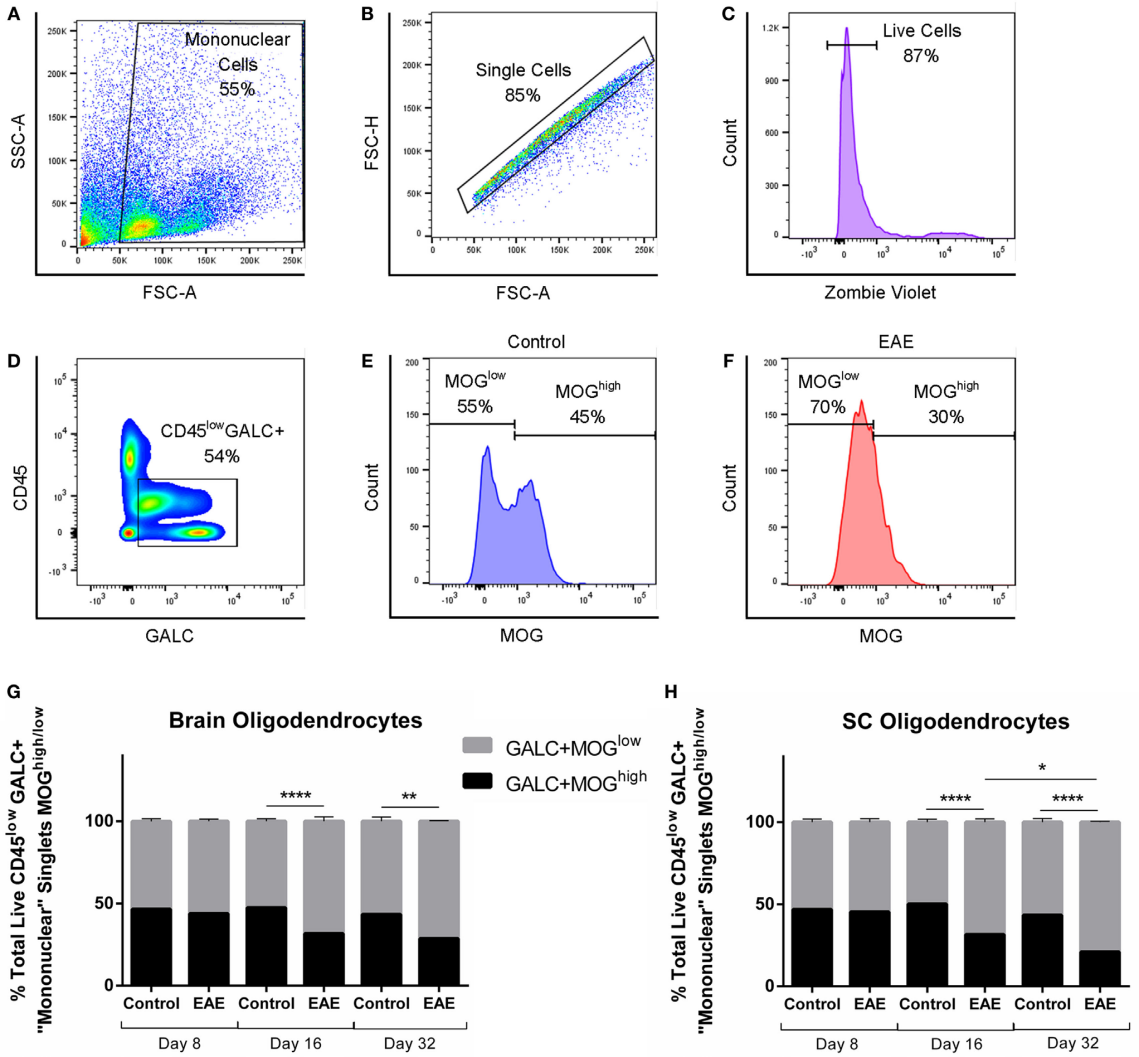
**The proportion of premyelinating to myelinating oligodendrocytes over the course of chronic EAE**. Flow cytometry to identify proportions of GALC + MOG^low^ premyelinating to GALC + MOG^high^ myelinating oligodendrocytes in the brain and spinal cord (SC) was carried out in EAE and control mice. Mononuclear cells were first gated **(A)**, followed by consecutive gating of singlets **(B)**, live cells **(C)**, and CD45^low^GALC+ cells for further analysis **(D)**. Representative histograms showing numbers of MOG^high^ cells and MOG^low^ cells in control mice **(E)** and EAE mice **(F)**. Bar graphs showing significant decreases in MOG^high^ myelinating, and subsequent significant increases in MOG^low^ premyelinating, oligodendrocytes in the brain **(G)**, and SC **(H)** of EAE mice compared to control mice at day 16 and 32 post-induction. No difference was seen between experimental groups preclinically at day 8. In the SC **(H)**, a significant decrease in MOG^high^ myelinating and subsequent significant increase in MOG^low^ premyelinating oligodendrocytes was also observed in EAE mice at day 32 compared to EAE mice at day 16. *P < 0.05, **P < 0.01, and ****P < 0.0001, two-way ANOVA followed by Tukey’s *post hoc* test, *n* = 3–5. Data are expressed as mean ± SEM.

Given the fact that oligodendrocyte damage in the absence of an innate or adaptive immune response is sufficient to confer central pain behaviors in mice (21), we next investigated whether oligodendrocyte changes are associated with the pain behaviors observed in our chronic EAE model. Flow cytometry was used to quantify changes in CNS myelination, as previously described (22). CD45^low^GALC + MOG^low^ premyelinating and CD45^low^GALC + MOG^high^ myelinating oligodendrocyte populations in the brain and SC were assessed over the course of chronic EAE. Mononuclear cells were first gated, followed by selection of live singlets (Figures 3A–C). CD45^low^ resident CNS cells positive for GALC were then gated (Figure 3D) and analyzed for changes in MOG positivity between control (Figure 3E) and EAE mice (Figure 3F).

No changes in myelination were found in the brain or SC at the preclinical stage of EAE (day 8 post-EAE induction, *n* = 5). At the clinical peak (day 16 post-induction), decreases in GALC+ MOG^high^ oligodendrocytes were seen, accompanied by a subsequent increase (*n* = 5, *p* < 0.0001) in the proportion of GALC+ MOG^low^ premyelinating oligodendrocytes in the brain (Figure 3G) and SC (Figure 3H). This was also apparent in the chronic phase of EAE (day 32 post-induction) in the brain (Figure 3G, *n* = 3, *p* < 0.01) and SC (Figure 3H, *n* = 3, *p* < 0.0001). Interestingly, despite the partial recovery typical of mice entering the chronic phase of EAE, this regain of hind limb movement was associated with progressive and continued loss of GALC+ MOG^high^ myelinating oligodendrocytes in the SC (Figure 3H, *n* = 3, *p* < 0.05).

The lack of a difference in oligodendrocyte subtypes in the preclinical stage suggests that CNS demyelination has not yet taken place at day 8 and does not correspond with the early emergence of allodynia in the sequelae of our EAE model.

### Peripheral and Central Changes in CD4+ Cell Infiltration in Mice with EAE

**Figure 4:**
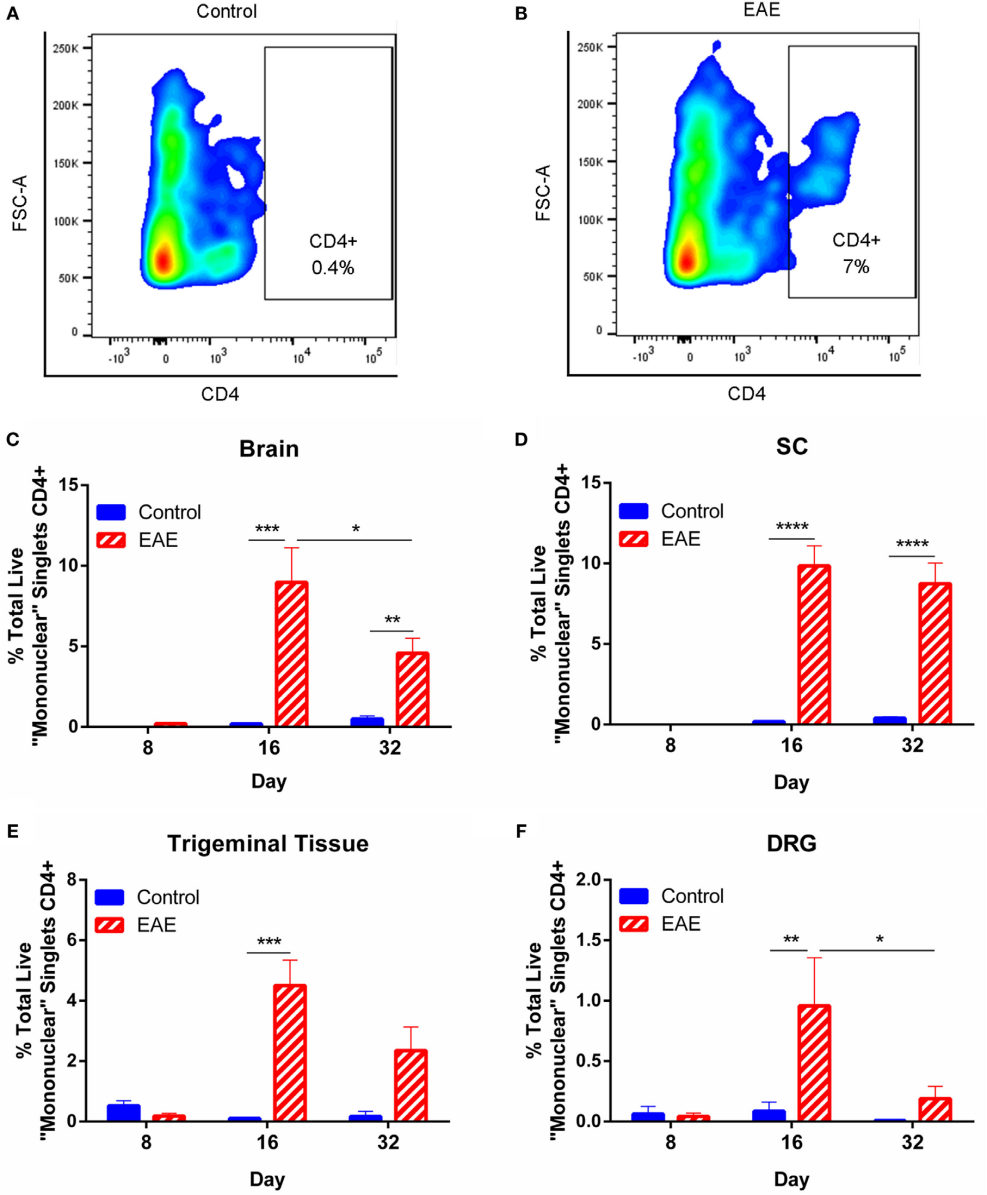
**CD4+ immune cell infiltration over the course of chronic EAE**. Flow cytometry to identify infiltrating CD4+ cells in the brain, spinal cord (SC), trigeminal tissue, and dorsal root ganglia (DRG) was carried out in EAE and control mice. Mononuclear cells were first gated, followed by singlets and live cells (as in Figures 3A–C). Representative contour plots from trigeminal tissue showing an absence of CD4+ cells in control animals **(A)** and the presence of CD4+ cells in EAE mice **(B)**. The percentage of CD4+ cells was significantly increased in the brain **(C)** and SC **(D)** of EAE mice compared to control mice at days 16 and 32 post-induction. The percentage of CD4+ cells was significantly reduced in the brain **(C)** of EAE mice at day 32 compared to EAE mice at day 16. The percentage of CD4+ cells was significantly increased in the trigeminal tissue **(E)** and in the DRG **(F)** of EAE mice compared to control mice at day 16 post-induction. In the DRG **(F)**, the percentage of CD4+ cells was significantly reduced in EAE mice at day 32 post-induction compared to EAE mice at day 16 post-induction. *P < 0.05, **P < 0.01, ***P < 0.001, and ****P < 0.0001, two-way ANOVA followed by Tukey’s *post hoc* test, *n* = 4–6. Data are expressed as mean ± SEM.

To investigate changes in neuroinflammation, we first assessed infiltration of cells expressing CD4 (a marker expressed predominantly on the surface of helper T cells as well as minor populations of other immune cells) into the CNS and peripheral ganglia over the course of chronic EAE using flow cytometry. Mononuclear cells were gated (as in Figure 3A) and single cells selected (as in Figure 3B), before gating viable cells (as in Figure 3C) for inclusion in further analysis for CD4 positivity in control and EAE mice (Figures 4A,B, respectively). Results showed that CD4+ cell infiltration was not apparent in the preclinical period, yet was clearly seen at the EAE clinical peak in all tissues analyzed including the brain, SC, trigeminal tissue, and DRG (Figures 4C–F, *n* = 4–6). CD4+ cells were also increased in the brain and SC in the chronic phase of the disease in EAE mice compared to control animals (Figures 4C,D, *n* = 6). A trend for increased CD4+ cells was seen in the trigeminal tissue of EAE mice compared to control animals in the chronic phase of disease, although this failed to reach significance (Figure 4E, *n* = 6, *p* = 0.0559). In the brain and DRG, CD4+ cells were reduced in the chronic phase compared to the EAE clinical peak (Figures 4C,F, *n* = 5–6). The absence of significant CD4+ cell infiltration at day 8 suggests that the preclinical allodynia observed in EAE mice is unlikely to arise from a central adaptive immune response.

### Peripheral and Central Changes in Macrophage and Glial Activation and T Cell Infiltration in Preclinical EAE

**Figure 5:**
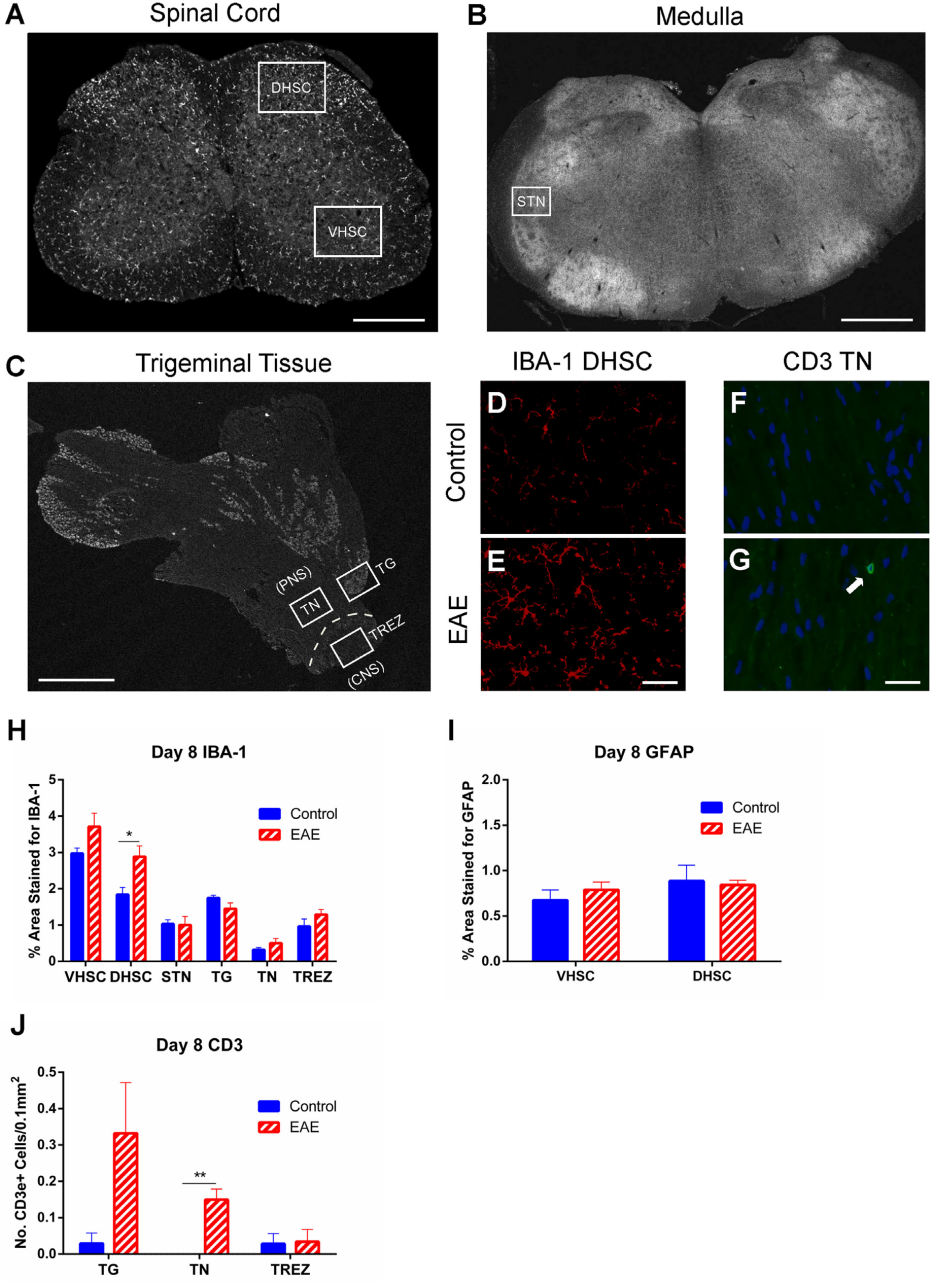
**Macrophage and glial activation and T cell infiltration in preclinical EAE**. IBA-1 and GFAP immunohistochemistry in the L3–5 spinal cord (SC), and IBA-1 and CD3 immunohistochemistry in the trigeminal afferent pathway were carried out on day 8 (preclinical EAE) in EAE and control mice. **(A)** Representative image of a section of L3–5 SC depicting areas of the ventral horn (VHSC) and dorsal horn (DHSC) selected for analysis. Scale bar equals 500 μm. **(B)** Representative image of a section of medulla showing the region corresponding to the spinal trigeminal nucleus (STN). Scale bar equals 1 mm. **(C)** Representative image depicting the peripheral trigeminal afferent pathway, inclusive of the trigeminal ganglia (TG), proximal trigeminal nerve (TN), and trigeminal root entry zone (TREZ). Scale bar equals 1 mm. Representative image of the DHSC taken from a control mouse **(D)** showing observably less IBA-1 staining than an EAE mouse **(E)**. Scale bar equals 50 μm. Representative image of a region of TN taken from a control mouse **(F)** showing an absence of CD3+ cells, which were present in EAE mice **(G)**. Scale bar equals 25 μm and white arrow indicates a CD3 immunoreactive cell with DAPI nuclear staining. **(H)** Bar graph summarizing levels of IBA-1 immunostaining, which were significantly increased in the DHSC of EAE mice; however, no difference was seen in any of the other tissues analyzed. **(I)** Bar graph depicting levels of GFAP immunostaining in the VHSC and DHSC, where no significant difference was seen between control and EAE mice. **(J)** Bar graph showing a significant increase in CD3+ cells in the TN, and a trend for increased numbers of CD3+ cells in the TG (*p* = 0.08). No difference was seen between experimental groups in the TREZ. *P < 0.05 and **P < 0.01, unpaired student’s *t*-test, *n* = 4–5. Data are expressed as mean ± SEM.

To determine whether an early response mediated by immune activation is associated with the preclinical allodynia observed in the hind paw and whisker pad, immunostaining for IBA-1 (macrophage/microglia marker) and GFAP (astrocyte marker) was carried out. Areas analyzed for IBA-1 included the dorsal (DHSC) and ventral (VHSC) horns of the L3–5 SC (Figure 5A), STN (Figure 5B), TG, TN, and TREZ (Figure 5C). There was no difference between EAE and control mice in IBA-1+ immunoreactivity in any of the tissue regions analyzed, except for an increase in IBA-1 expressing cells in the dorsal horn of the SC of EAE mice compared to control mice (Figures 5D,E,H, *n* = 4–5, *p* < 0.05). Analysis of GFAP expression in the dorsal and ventral horns of the SC revealed no differences in astrocyte activation between EAE and control mice (Figure 5I, *n* = 4–5).

Since flow cytometric analysis of the CNS and peripheral ganglia showed no increase in CD4+ cells at day 8 (Figure 4), immunostaining for CD3+ T cells was carried out with a focus on specific regions of the trigeminal afferent pathway (Figure 5C). This was done to ascertain whether the preclinical facial allodynia of EAE mice may be linked to a subtle early T cell infiltration into the peripheral trigeminal afferent pathway occurring prior to more overt central infiltration. A small but significant increase in CD3+ cells was found restricted to the TN (Figures 5F,G,J, *n* = 4–5, *p* < 0.01), and a similar trend, though not statistically significant, was seen in the TG (Figure 5J, *n* = 4–5).

### Peripheral and Central Changes in Macrophage and Glial Activation and T Cell Infiltration in Clinical EAE

**Figure 6:**
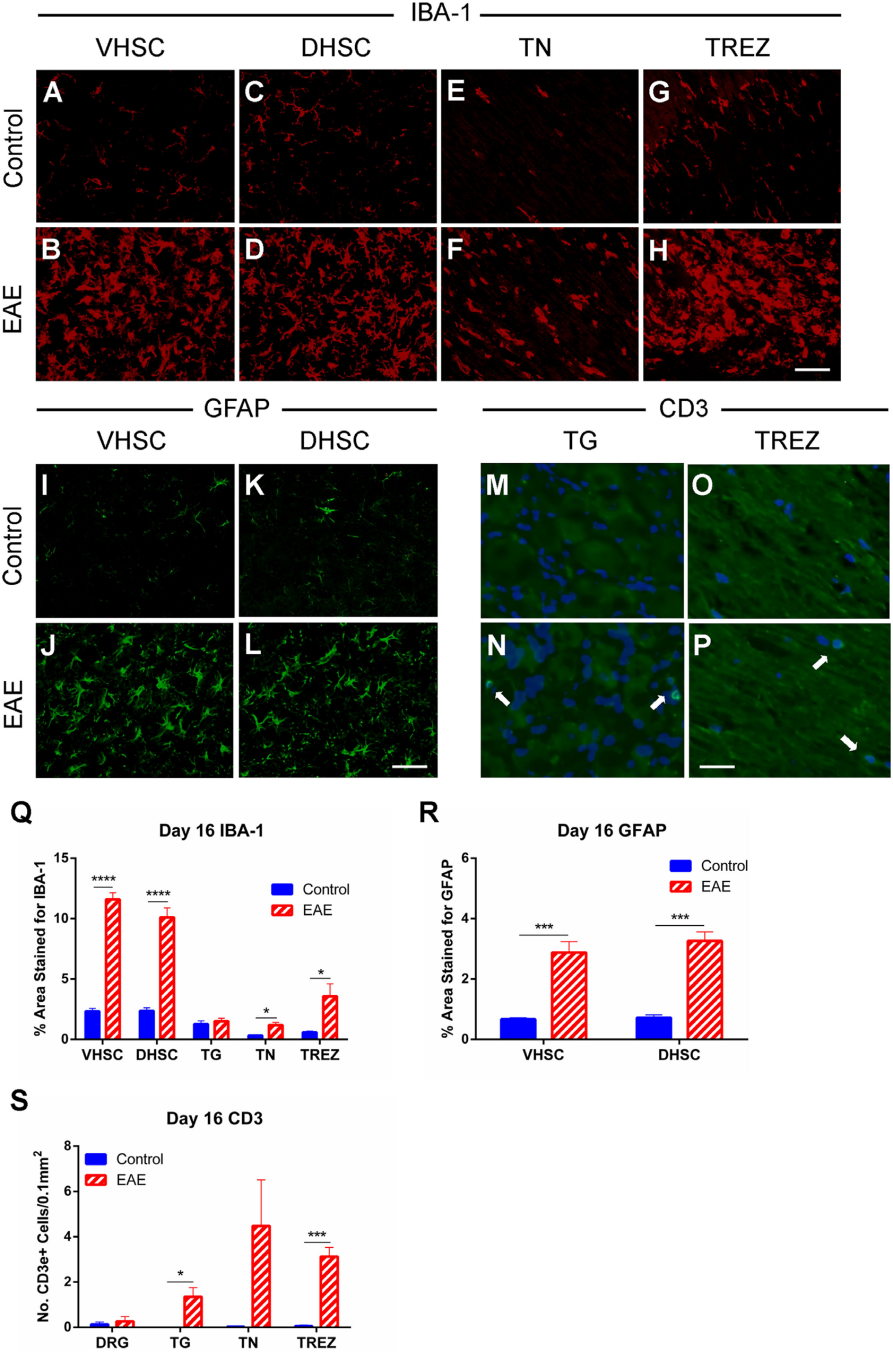
**Macrophage and glial activation and T cell infiltration in clinical EAE**. IBA-1 and GFAP immunohistochemistry in the L3–5 spinal cord (SC), IBA-1, and CD3 immunohistochemistry in the trigeminal afferent pathway and CD3 immunohistochemistry in the L3–5 dorsal root ganglia (DRG) were carried out on day 16 (at the EAE clinical peak) in EAE and control mice. Representative images showing less IBA-1 immunostaining in the ventral horn of the spinal cord (VHSC) **(A)**, dorsal horn of the spinal cord (DHSC) **(C)**, trigeminal nerve (TN) **(E)**, and trigeminal root entry zone (TREZ) **(G)** of control mice compared to the VHSC **(B)**, DHSC **(D)**, TN **(F)**, and TREZ **(H)** of EAE mice. Representative images showing less GFAP immunostaining in the VHSC **(I)** and DHSC **(K)** of control mice compared to VHSC **(J)** and DHSC **(L)** of EAE mice. Representative images showing an absence of CD3+ cells in the trigeminal ganglia (TG) **(M)** and TREZ **(O)** of control mice, which were visible in TG **(N)** and TREZ **(P)** of EAE mice. Scale bar equals 25 μm and white arrows indicate CD3 immunoreactive cells with DAPI nuclear staining. **(Q)** Bar graph showing significantly increased levels of IBA-1 immunostaining in the VHSC, DHSC, TN, and TREZ in EAE mice compared to control mice. No difference was seen in the TG between experimental groups. **(R)** Bar graph showing significantly increased levels of GFAP immunostaining in both the VHSC and DHSC in EAE mice compared to control mice. **(S)** Bar graph showing no difference in CD3+ cell numbers in the DRG, but significant increases in the TG and TREZ, as well as a trend for increased levels in the TN (*p* = 0.095) in EAE compared to control mice. *P < 0.05, ***P < 0.001, and ****P < 0.0001, unpaired student’s *t*-test, *n* = 4–5. Data are expressed as mean ± SEM.

Immunohistochemical analysis to characterize changes in activation of macrophages/microglia in the SC and trigeminal afferent pathway and astrocytes in the SC at the clinical peak of EAE was also conducted. A substantial increase (*n* = 4–5, *p* < 0.0001) in IBA-1 expressing cells in both the ventral (Figures 6A,B,Q) and dorsal horns (Figures 6C,D,Q) of the SC in EAE mice compared to control mice was found. An increase in IBA-1 immunoreactivity was also apparent in the TN (Figures 6E,F,Q, *n* = 4–5, *p* < 0.05) and TREZ (Figures 6G,H,Q, *n* = 4–5, *p* < 0.05). GFAP+ cells were similarly increased in the ventral (Figures 6I,J,R, *n* = 4–5, *p* < 0.001) and dorsal (Figures 6K,L,R, *n* = 4–5, *p* < 0.001) horns of the SC in EAE mice compared to control mice.

Since T cell infiltration into the CNS at the EAE clinical peak has been demonstrated by numerous studies and by our flow cytometric analysis (Figure 4), we carried out immunohistochemistry for CD3 in the trigeminal afferent pathway and L3–5 DRGs. We found CD3+ cells in increased numbers within the TG (Figures 6M,N,S, *n* = 4–5, *p* < 0.05) and TREZ (Figures 6O,P,S, *n* = 4–5, *p* < 0.001) in EAE mice compared to control mice. A trend for increased CD3+ cells in the TN was also found, although this failed to reach significance (Figure 6S, *n* = 4–5). Immunostaining for CD3+ cells in the DRG showed no significant difference in T cell numbers between EAE and control mice (Figure 6S, *n* = 4–5).

**Figure 7:**
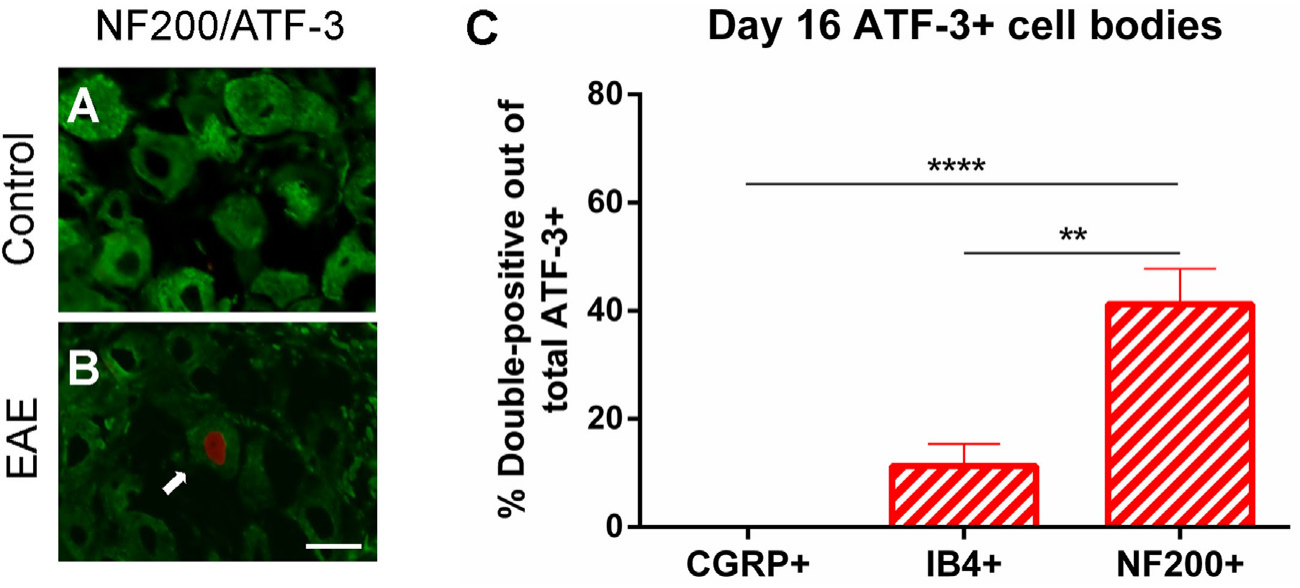
**Specific damage to myelinated A-class fibers in clinical EAE**. Representative images of NF200/ATF-3 double-labeling showing the presence of NF200+ATF-3+ cells in the trigeminal ganglion of EAE mice **(B)**, but not control mice **(A)** at day 16. Scale bar equals 25 μm and white arrow indicates an NF200 immunoreactive cell with ATF-3 nuclear staining. **(C)** Bar graph showing significantly increased numbers of NF200+ATF-3+ cell bodies compared to CGRP+ATF-3+ and IB4+ATF-3+ cell bodies in the trigeminal ganglia of EAE mice. **P < 0.01 and ****P < 0.0001, one-way ANOVA followed by Tukey’s *post hoc* test, *n* = 4–5. Data are expressed as mean ± SEM.

As significant changes in T cell infiltration were observed in the TG in clinical EAE (day 16), we next examined whether ATF-3 (a marker of neurons with damaged primary afferents) is induced in peptidergic (CGRP+) and non-peptidergic (IB4+) small diameter neurons, and NF200+ large diameter myelinated neurons using immunohistochemistry. This was of interest as injured DRG neurons have been linked to pain behaviors in mice with peripheral nerve injury (23) and rats with brachial plexus avulsion (24). We found a small, though significant, increase in the percentage of total ATF-3 immunopositive cell bodies in the TG of EAE (1% ± 0.2) compared to control (0.1% ± 0.05) mice (Unpaired Student’s *t*-test, *n* = 4–5, *p* < 0.01). Further analysis revealed ATF3+ neurons present in EAE mice (Figure 7B), but not controls (Figure 7A), predominantly co-expressed NF200. An increase in NF200+ATF-3+ cell bodies compared to CGRP+ATF-3+ (*n* = 4–5, *p* < 0.0001), and IB4+ATF-3+ (*n* = 4–5, *p* < 0.01) cell bodies was seen in the TG of mice at the clinical peak of EAE (Figure 7C) suggesting that neuronal damage is selective for myelinated NF200+ neurons.

## Discussion

Although it is well established that neuroinflammation and demyelination are important contributors to pain behaviors in MS and EAE (25, 26), the relationship between the pain phenotypes at different stages of disease and potential peripheral and central contributors is unclear. We found that stimulus-evoked and spontaneous pain in EAE may derive from unique pathological mechanisms, with the former arising in the absence of overt central lesion formation. A subtle increase in IBA-1+ cell numbers was seen in the dorsal horn of the SC in preclinical disease, and this corresponded to increased allodynia of the hind paw. Analysis of the trigeminal afferent pathway revealed that facial allodynia coincided with peripheral changes including a small, but significant, infiltration of T cells into the TN during preclinical disease and specific damage to myelinated NF200+ neurons at the EAE clinical peak. Clinical EAE and the onset of spontaneous pain appear to be associated with more overt changes, with potential peripheral and central contributions.

This study was conducted in female mice to account for the fact that MS is universally more prevalent in women than men (27). We show here that female mice display both stimulus-evoked and non-evoked pain behaviors throughout the course of chronic EAE, and that facial allodynia and facial grimacing predominate in unique stages of the disease. This finding suggests different underlying etiologies for EAE-induced evoked pain and spontaneous pain. Similarly to Thorburn and colleagues (15), we describe facial allodynia and trigeminal pathology in mice with EAE as described in patients suffering from trigeminal neuralgia associated with MS (28–31). In addition to effectively modeling trigeminal neuralgia, the use of von Frey filaments to elicit facial allodynia allows continual testing of cutaneous hypersensitivity during periods of otherwise confounding lower body motor impairment. Interestingly, the development of facial and hind paw allodynia arose prior to the onset of clinical symptoms, in line with a previous study that showed both mechanical and cold allodynia of the hind paw in the preclinical period of EAE (10). We also present the utility of the MGS (20) in measuring spontaneous pain over the course of chronic EAE, which may increase the translational capacity of pain research in EAE when testing analgesic therapies for MS. Facial palsy occurs in some patients with MS (32) and may be due to peripheral lesions affecting motor facial nerve fibers or central lesions in the area of the facial nucleus (33). While EAE is typically considered a central demyelinating disorder, we cannot exclude the possibility that increased facial grimacing may be influenced by demyelination of lower motor neurons resulting in brainstem reflex abnormalities and a compromised ability of EAE mice to exhibit facial expressions. Regardless, such demyelination is also known to be a major contributor to neuropathic pain (34), and therefore does not discount the interpretation of increased facial grimacing as a response to pain. Additionally, it remains unclear whether the facial grimacing observed in EAE mice is reflective of spontaneous pain similar to trigeminal neuralgia or of spontaneous pain arising in area(s) unrelated to the face. As the MGS was reported to be non-useful for measuring neuropathic pain in peripheral nerve injury models (20), we cannot discount that the facial grimacing induced in EAE mice may be due to nociceptive pain arising as a consequence of musculoskeletal problems secondary to lower body paralysis and spasticity. Given that the majority of MS patients with central pain also suffer concurrent paresis (35), it is perhaps desirable to measure pain in stages of EAE where lower body motor impairment is apparent as has been made possible using the methods described in the current study.

A recent study demonstrated that conditional ablation of oligodendrocytes leads to the development of pain behaviors in mice. This suggests that a loss of oligodendrocyte function, which maintains axonal integrity in the CNS, is able to trigger the development of neuropathic pain (21). We therefore assessed demyelination over the course of chronic EAE using flow cytometry. Although we found decreases in GALC+ MOG^high^ myelinating oligodendrocytes in the brain and SC in the peak and chronic phases of clinical EAE, no changes were observed in the preclinical stage. Accompanying these clinical decreases in myelination was a subsequent increase in GALC + MOG^low^ premyelinating oligodendrocytes. This suggests an arrest of oligodendrocyte maturation at the premyelinating stage, presumably due to the presence of autoreactive T cells sensitized to the MOG antigen. Since overt demyelination was only observed in periods of clinical EAE, it appears that allodynia, which predominates during the preclinical phase of the disease, is not directly related to oligodendrocyte dysfunction or damage.

Since previous studies have purported links between central lesions in areas such as the SC (9, 10, 12, 36) and trigeminal nuclei (15) with evoked pain behaviors, we next analyzed the CNS of mice with preclinical EAE for T cell infiltration and glial changes. No increase in CD4+ cells was observed in the brain or SC in the preclinical period, which suggests that a central adaptive immune response does not mediate allodynia in preclinical EAE. Glial activation in the dorsal horn of the SC and STN was also analyzed, and a subtle, yet statistically significant, increase in IBA-1-expressing cells in the dorsal horn of the SC was seen. Increased numbers of IBA-1+ cells have previously been shown in the primary somatosensory cortex in preclinical EAE (16), and perivascular microglial clustering in the SC occurs prior to the onset of clinical symptoms and demyelination. This clustering was shown to correlate with fibrinogen deposition in the SC, which was taken as a reflection of early blood–brain barrier disruption (37). It is unknown whether the increase in IBA-1 expressing cells in the dorsal horn was provoked by perivascular microglial clustering in the present study, and the use of chimeric mice (38–41) or a marker specific for microglia (42) would allow for clarification. Microglia have been linked to the initiation of mechanical allodynia in a range of chronic pain models (43–47). In particular, after nerve damage, spinal microglia have been shown to change their morphology, phenotype, and motility, express intracellular signaling molecules (for example, p38 mitogen-activated protein kinase), and release brain-derived neurotrophic factor, cytokines such as tumor necrosis factor (TNF) and interleukin (IL)-1β, and chemokines, thus potentiating aberrant nociceptive signaling (48–50). It is therefore feasible that microglial activation in the L3–5 dorsal horn may have contributed to the mechanical allodynia of the hind paw seen in this study. However, a similar increase in IBA-1 expressing cells was not apparent in the STN where small diameter fibers (C and Aδ) carrying information concerning pain and temperature terminate. A more comprehensive analysis inclusive of all brainstem trigeminal nuclei would be useful to elucidate whether an increase in IBA-1+ cells similar to what is seen in the dorsal horn of the SC is apparent in these areas, and whether this may play a role in the development of facial allodynia in preclinical EAE. It is also interesting to note that different immune cells have recently been shown to mediate neuropathic pain behavior following peripheral nerve injury in male and female mice. While spinal microglia mediated the development of mechanical pain hypersensitivity in male mice, T cells appeared to be responsible for such pain in female mice (51) suggesting sexual dimorphism in pain processing.

Given that allodynia appears to arise in the absence of distinct central lesion formation, we next sought to ascertain whether changes in the PNS are associated with stimulus-evoked pain in EAE. In analyzing the trigeminal afferent pathway during preclinical EAE (day 8), we found that facial allodynia coincided with T cell infiltration into the TN. Considering we studied MOG_35–55_-induced EAE, the antigen specificity of the T cells infiltrating the TN, where the MOG antigen is not expressed, remains unknown. The clinical peak of EAE is associated with central neuroinflammation. T cells are known to infiltrate into the CNS (52) and trigeminal afferent pathway including the TG, TN, and TREZ (15) in EAE, and this was confirmed in the present study. Infiltrating CD4+ cells in EAE have previously been shown to be pathogenic and primarily of a Th1 and Th17 phenotype (53, 54), both of which have been implicated in the generation of neuropathic pain through the production of their signature cytokines IFN-γ (55, 56) and IL-17 (57, 58). T cell infiltration along with colocalized glial activation in the superficial dorsal horn (10) and trigeminal afferent pathway (15) were postulated to contribute to the development of EAE-induced evoked hind paw and facial hypersensitivity, respectively. Although pain hypersensitivity developed prior to significant central neuroinflammation, our observation of a substantial infiltration of CD4+ cells in the CNS, microglia, and astrocyte activation in the dorsal horn of the SC, and an increased T cell infiltration, and macrophage/microglial activation in multiple areas along the trigeminal afferent pathway may have contributed to pain behaviors during EAE clinical peak. In parallel with another recent study (17), we found increased numbers of infiltrating CD4+ cells in the DRG in clinical EAE, where it has been previously reported that rats with EAE have elevated expression of fractalkine (CX3CL1) and its receptor (CX3CR1) (59), and increased levels of TNF-α (60, 61), and IL-1β (62). Innate immune cells such as NK cells, neutrophils, and mast cells are also known to be involved in the pathogenesis of EAE (63–65); however, their role in pain associated with EAE and MS is unclear. Mast cells in particular have been implicated in the production of neuropathic pain in models of peripheral nerve injury (66) and chemotherapy-induced peripheral neuropathy (67). An expanded characterization of cells such as these in the EAE model and their relationship to pain behaviors would be an important addition to the current knowledge.

The facial allodynia seen in the clinical period of EAE was associated with neuronal damage in myelinated A-class sensory fibers, but not in unmyelinated C-fibers in the TG. ATF-3 expression has also been recently shown in a mixed population of sensory neurons in EAE, including those with myelinated axons (NF200+) and in others that express TRPV1 (17). Previous studies in models of monoarthritic joint pain (68) and capsaicin-induced pain (69) have shown that damage to cell bodies in the DRG, as indicated by ATF-3 staining, primarily involves CGRP+ peptidergic and IB4+ non-peptidergic C-fibers. EAE is a demyelinating disease, and this could account for why myelinated sensory fibers are injured while C-fibers appear largely preserved. Large myelinated Aβ fibers are implicated in the dorsal horn circuit responsible for producing mechanical allodynia (70, 71). Indeed, blockade of large diameter NF200+ A-class sensory fibers, but not C-fibers, abrogates mechanical allodynia in a range of neuropathic pain models including chemotherapy-induced peripheral neuropathy, nerve injury, and diabetic neuropathy (72). Further studies addressing whether Aβ fibers are specifically damaged in EAE, and whether this occurs in the dorsal horn circuit as well as the trigeminal afferent pathway would help to elucidate the etiology of allodynia in MS.

The observation that the facial allodynia and grimacing observed in the current study predominate in unique clinical phases of EAE is an interesting one. Facial allodynia develops in the absence of overt central changes but rather corresponds with a subtle peripheral change of a small, yet significant, infiltration of T cells into the TN. Facial allodynia appears to quite sharply decrease following the onset of EAE compared to the preclinical period. While it is presently unknown why this occurs, hypoesthesia is also common in MS (35), and diminished responses to subcutaneous formalin injection in EAE have been linked to dysregulation of the glutamatergic system (73). The mechanisms underlying the facial grimacing observed in clinical EAE are likely to be multifactorial as the behavior coincides with T cell infiltration into the CNS, trigeminal afferent pathway and DRG, as well as an increase in IBA-1+ cell numbers and specific damage to large myelinated neurons in the trigeminal afferent pathway, gliosis in the dorsal horn of the SC, and central demyelination. Although the precise etiology of facial allodynia and grimacing in EAE remains unclear, both methods provide an invaluable approach to better understand the mechanisms involved in the production of pain in EAE.

In summary, we observed different pain phenotypes including stimulus-evoked and spontaneous pain predominating in unique stages of the chronic EAE model, in accordance with the diverse pain phenotypes seen in MS patients (6). The pain behaviors we observed were associated with several neuroinflammatory changes in both the peripheral nervous system and CNS and are likely to involve numerous underlying mechanisms.

## Author Contributions

SD designed experiments, performed animal immunizations, behavioral tests, flow cytometry, immunohistochemistry, microscopy, image analysis, and drafted the manuscript. CP assisted with behavioral testing and immunohistochemistry. PM was involved in tissue dissection and assisted with image analysis. JL and PC assisted in experiment design and interpretation of data. GM-T conceived and designed the study, assisted in interpretation of data, and critically revised the manuscript. All authors read and approved the manuscript.

## Conflict of Interest Statement

The authors declare that the research was conducted in the absence of any commercial or financial relationships that could be construed as a potential conflict of interest.

## References

[1] Noseworthy JH Lucchinetti C Rodriguez M Weinshenker BG Multiple sclerosis. N Engl J Med (2000) 343:938–52. 10.1056/NEJM200009283431307 11006371

[2] Hauser SL Oksenberg JR. The neurobiology of multiple sclerosis: genes, inflammation, and neurodegeneration. Neuron (2006) 52:61–76. 10.1016/j.neuron.2006.09.011 17015227

[3] Ebers GC Environmental factors and multiple sclerosis. Lancet Neurol (2008) 7:268–77. 10.1016/S1474-4422(08)70042-5 18275928

[4] Drulovic J Basic-Kes V Grgic S Vojinovic S Dincic E Toncev G The prevalence of pain in adults with multiple sclerosis: a multicenter cross-sectional survey. Pain Med (2015) 16:1597–602. 10.1111/pme.12731 26087108

[5] Svendsen KB Jensen TS Hansen HJ Bach FW. Sensory function and quality of life in patients with multiple sclerosis and pain. Pain (2005) 114:473–81. 10.1016/j.pain.2005.01.015 15777872

[6] Solaro C Uccelli MM. Management of pain in multiple sclerosis: a pharmacological approach. Nat Rev Neurol (2011) 7:519–27. 10.1038/nrneurol.2011.120 21844896

[7] Truini A Barbanti P Pozzilli C Cruccu G. A mechanism-based classification of pain in multiple sclerosis. J Neurol (2013) 260:351–67. 10.1007/s00415-012-6579-2 22760942 PMC3566383

[8] Duffy SS Lees JG Moalem-Taylor G. The contribution of immune and glial cell types in experimental autoimmune encephalomyelitis and multiple sclerosis. Mult Scler Int (2014) 2014:285245. 10.1155/2014/285245 25374694 PMC4211315

[9] Aicher SA Silverman MB Winkler CW Bebo BF Jr. Hyperalgesia in an animal model of multiple sclerosis. Pain (2004) 110:560–70. 10.1016/j.pain.2004.03.025 15288396

[10] Olechowski CJ Truong JJ Kerr BJ. Neuropathic pain behaviours in a chronic-relapsing model of experimental autoimmune encephalomyelitis (EAE). Pain (2009) 141:156–64. 10.1016/j.pain.2008.11.002 19084337

[11] Rodrigues DH Sachs D Teixeira AL. Mechanical hypernociception in experimental autoimmune encephalomyelitis. Arq Neuropsiquiatr (2009) 67:78–81. 10.1590/S0004-282X2009000100019 19330217

[12] Lu J Kurejova M Wirotanseng LN Linker RA Kuner R Tappe-Theodor A. Pain in experimental autoimmune encephalitis: a comparative study between different mouse models. J Neuroinflammation (2012) 9:233. 10.1186/1742-2094-9-233 23039175 PMC3582444

[13] Yuan S Shi Y Tang SJ. Wnt signaling in the pathogenesis of multiple sclerosis-associated chronic pain. J Neuroimmune Pharmacol (2012) 7:904–13. 10.1007/s11481-012-9370-3 22547300

[14] Schmitz K Pickert G Wijnvoord N Haussler A Tegeder I. Dichotomy of CCL21 and CXCR3 in nerve injury-evoked and autoimmunity-evoked hyperalgesia. Brain Behav Immun (2013) 32:186–200. 10.1016/j.bbi.2013.04.011 23643685

[15] Thorburn KC Paylor JW Webber CA Winship IR Kerr BJ Facial hypersensitivity and trigeminal pathology in mice with experimental autoimmune encephalomyelitis (EAE). Pain (2016) 157(3):627–42. 10.1097/j.pain.0000000000000409 26545087

[16] Potter LE Paylor JW Suh JS Tenorio G Caliaperumal J Colbourne F Altered excitatory-inhibitory balance within somatosensory cortex is associated with enhanced plasticity and pain sensitivity in a mouse model of multiple sclerosis. J Neuroinflammation (2016) 13:142. 10.1186/s12974-016-0609-4 27282914 PMC4901403

[17] Frezel N Sohet F Daneman R Basbaum AI Braz JM. Peripheral and central neuronal ATF3 precedes CD4+ T-cell infiltration in EAE. Exp Neurol (2016) 283:224–34. 10.1016/j.expneurol.2016.06.019 27343802 PMC5500277

[18] Dixon WJ The up-and-down method for small samples. J Am Stat Assoc (1965) 60:967–78. 10.1080/01621459.1965.10480843

[19] Lyons DN Kniffin TC Zhang LP Danaher RJ Miller CS Bocanegra JL Trigeminal inflammatory compression (TIC) injury induces chronic facial pain and susceptibility to anxiety-related behaviors. Neuroscience (2015) 295:126–38. 10.1016/j.neuroscience.2015.03.051 25818051 PMC4408265

[20] Langford DJ Bailey AL Chanda ML Clarke SE Drummond TE Echols S Coding of facial expressions of pain in the laboratory mouse. Nat Methods (2010) 7:447–9. 10.1038/nmeth.1455 20453868

[21] Gritsch S Lu J Thilemann S Wortge S Mobius W Bruttger J Oligodendrocyte ablation triggers central pain independently of innate or adaptive immune responses in mice. Nat Commun (2014) 5:5472. 10.1038/ncomms6472 25434649 PMC4268702

[22] Robinson AP Rodgers JM Goings GE Miller SD. Characterization of oligodendroglial populations in mouse demyelinating disease using flow cytometry: clues for MS pathogenesis. PLoS One (2014) 9:e107649. 10.1371/journal.pone.0107649 25247590 PMC4172589

[23] Obata K Yamanaka H Fukuoka T Yi D Tokunaga A Hashimoto N Contribution of injured and uninjured dorsal root ganglion neurons to pain behavior and the changes in gene expression following chronic constriction injury of the sciatic nerve in rats. Pain (2003) 101:65–77. 10.1016/S0304-3959(02)00296-8 12507701

[24] Matsuura Y Ohtori S Iwakura N Suzuki T Kuniyoshi K Takahashi K. Expression of activating transcription factor 3 (ATF3) in uninjured dorsal root ganglion neurons in a lower trunk avulsion pain model in rats. Eur Spine J (2013) 22:1794–9. 10.1007/s00586-013-2733-5 23471575 PMC3731502

[25] Solaro C Trabucco E Messmer Uccelli M. Pain and multiple sclerosis: pathophysiology and treatment. Curr Neurol Neurosci Rep (2013) 13:320. 10.1007/s11910-012-0320-5 23250765

[26] Khan N Smith MT. Multiple sclerosis-induced neuropathic pain: pharmacological management and pathophysiological insights from rodent EAE models. Inflammopharmacology (2014) 22:1–22. 10.1007/s10787-013-0195-3 24234347 PMC3933737

[27] Harbo HF Gold R Tintore M. Sex and gender issues in multiple sclerosis. Ther Adv Neurol Disord (2013) 6:237–48. 10.1177/1756285613488434 23858327 PMC3707353

[28] Love S Gradidge T Coakham HB. Trigeminal neuralgia due to multiple sclerosis: ultrastructural findings in trigeminal rhizotomy specimens. Neuropathol Appl Neurobiol (2001) 27:238–44. 10.1046/j.0305-1846.2001.00318.x 11489143

[29] Nakashima I Fujihara K Kimpara T Okita N Takase S Itoyama Y. Linear pontine trigeminal root lesions in multiple sclerosis: clinical and magnetic resonance imaging studies in 5 cases. Arch Neurol (2001) 58:101–4. 10.1001/archneur.58.1.101 11176942

[30] Cruccu G Biasiotta A Di Rezze S Fiorelli M Galeotti F Innocenti P Trigeminal neuralgia and pain related to multiple sclerosis. Pain (2009) 143:186–91. 10.1016/j.pain.2008.12.026 19171430

[31] Swinnen C Lunskens S Deryck O Casselman J Vanopdenbosch L MRI characteristics of trigeminal nerve involvement in patients with multiple sclerosis. Mult Scler Relat Disord (2013) 2:200–3. 10.1016/j.msard.2012.12.002 25877726

[32] Fukazawa T Moriwaka F Hamada K Hamada T Tashiro K. Facial palsy in multiple sclerosis. J Neurol (1997) 244:631–3. 10.1007/s004150050158 9402539

[33] Gilden DH Clinical practice. Bell’s Palsy. N Engl J Med (2004) 351:1323–31. 10.1056/NEJMcp041120 15385659

[34] Wallace VC Cottrell DF Brophy PJ Fleetwood-Walker SM. Focal lysolecithin-induced demyelination of peripheral afferents results in neuropathic pain behavior that is attenuated by cannabinoids. J Neurosci (2003) 23:3221–33. 12716929 10.1523/JNEUROSCI.23-08-03221.2003 PMC6742302

[35] Osterberg A Boivie J Thuomas KA Central pain in multiple sclerosis – prevalence and clinical characteristics. Eur J Pain (2005) 9:531–42. 10.1016/j.ejpain.2004.11.005 16139182

[36] Olechowski CJ Tenorio G Sauve Y Kerr BJ. Changes in nociceptive sensitivity and object recognition in experimental autoimmune encephalomyelitis (EAE). Exp Neurol (2013) 241:113–21. 10.1016/j.expneurol.2012.12.012 23291347

[37] Davalos D Ryu JK Merlini M Baeten KM Le Moan N Petersen MA Fibrinogen-induced perivascular microglial clustering is required for the development of axonal damage in neuroinflammation. Nat Commun (2012) 3:1227. 10.1038/ncomms2230 23187627 PMC3514498

[38] Nakano K Migita M Mochizuki H Shimada T. Differentiation of transplanted bone marrow cells in the adult mouse brain. Transplantation (2001) 71:1735–40. 10.1097/00007890-200106270-00006 11455251

[39] Vallieres L Sawchenko PE. Bone marrow-derived cells that populate the adult mouse brain preserve their hematopoietic identity. J Neurosci (2003) 23:5197–207. 12832544 10.1523/JNEUROSCI.23-12-05197.2003 PMC6741180

[40] Mizutani M Pino PA Saederup N Charo IF Ransohoff RM Cardona AE The fractalkine receptor but not CCR2 is present on microglia from embryonic development throughout adulthood. J Immunol (2012) 188:29–36. 10.4049/jimmunol.1100421 22079990 PMC3244524

[41] Garcia JA Cardona SM Cardona AE. Analyses of microglia effector function using CX3CR1-GFP knock-in mice. Methods Mol Biol (2013) 1041:307–17. 10.1007/978-1-62703-520-0_27 23813389 PMC3980416

[42] Butovsky O Jedrychowski MP Moore CS Cialic R Lanser AJ Gabriely G Identification of a unique TGF-beta-dependent molecular and functional signature in microglia. Nat Neurosci (2014) 17:131–43. 10.1038/nn0914-1286d 24316888 PMC4066672

[43] Svensson CI Marsala M Westerlund A Calcutt NA Campana WM Freshwater JD Activation of p38 mitogen-activated protein kinase in spinal microglia is a critical link in inflammation-induced spinal pain processing. J Neurochem (2003) 86:1534–44. 10.1046/j.1471-4159.2003.01969.x 12950462

[44] Tsuda M Shigemoto-Mogami Y Koizumi S Mizokoshi A Kohsaka S Salter MW P2X4 receptors induced in spinal microglia gate tactile allodynia after nerve injury. Nature (2003) 424:778–83. 10.1038/nature01786 12917686

[45] Raghavendra V Tanga FY DeLeo JA. Complete Freunds adjuvant-induced peripheral inflammation evokes glial activation and proinflammatory cytokine expression in the CNS. Eur J Neurosci (2004) 20:467–73. 10.1111/j.1460-9568.2004.03514.x 15233755

[46] Inoue K Tsuda M. Microglia and neuropathic pain. Glia (2009) 57:1469–79. 10.1002/glia.20871 19306358

[47] Tsuda M Beggs S Salter MW Inoue K. Microglia and intractable chronic pain. Glia (2013) 61:55–61. 10.1002/glia.22379 22740331

[48] Jin SX Zhuang ZY Woolf CJ Ji RR. p38 mitogen-activated protein kinase is activated after a spinal nerve ligation in spinal cord microglia and dorsal root ganglion neurons and contributes to the generation of neuropathic pain. J Neurosci (2003) 23:4017–22. 12764087 10.1523/JNEUROSCI.23-10-04017.2003 PMC6741086

[49] Ulmann L Hatcher JP Hughes JP Chaumont S Green PJ Conquet F Up-regulation of P2X4 receptors in spinal microglia after peripheral nerve injury mediates BDNF release and neuropathic pain. J Neurosci (2008) 28:11263–8. 10.1523/JNEUROSCI.2308-08.2008 18971468 PMC6671487

[50] Grace PM Hutchinson MR Maier SF Watkins LR. Pathological pain and the neuroimmune interface. Nat Rev Immunol (2014) 14:217–31. 10.1038/nri3621 24577438 PMC5525062

[51] Sorge RE Mapplebeck JC Rosen S Beggs S Taves S Alexander JK Different immune cells mediate mechanical pain hypersensitivity in male and female mice. Nat Neurosci (2015) 18:1081–3. 10.1038/nn.4053 26120961 PMC4772157

[52] Fletcher JM Lalor SJ Sweeney CM Tubridy N Mills KH. T cells in multiple sclerosis and experimental autoimmune encephalomyelitis. Clin Exp Immunol (2010) 162:1–11. 10.1111/j.1365-2249.2010.04143.x 20682002 PMC2990924

[53] Jager A Dardalhon V Sobel RA Bettelli E Kuchroo VK. Th1, Th17, and Th9 effector cells induce experimental autoimmune encephalomyelitis with different pathological phenotypes. J Immunol (2009) 183:7169–77. 10.4049/jimmunol.0901906 19890056 PMC2921715

[54] Murphy AC Lalor SJ Lynch MA Mills KH. Infiltration of Th1 and Th17 cells and activation of microglia in the CNS during the course of experimental autoimmune encephalomyelitis. Brain Behav Immun (2010) 24:641–51. 10.1016/j.bbi.2010.01.014 20138983

[55] Costigan M Moss A Latremoliere A Johnston C Verma-Gandhu M Herbert TA T-cell infiltration and signaling in the adult dorsal spinal cord is a major contributor to neuropathic pain-like hypersensitivity. J Neurosci (2009) 29:14415–22. 10.1523/JNEUROSCI.4569-09.2009 19923276 PMC2813708

[56] Tsuda M Masuda T Kitano J Shimoyama H Tozaki-Saitoh H Inoue K. IFN-gamma receptor signaling mediates spinal microglia activation driving neuropathic pain. Proc Natl Acad Sci U S A (2009) 106:8032–7. 10.1073/pnas.0810420106 19380717 PMC2683100

[57] Kim CF Moalem-Taylor G. Interleukin-17 contributes to neuroinflammation and neuropathic pain following peripheral nerve injury in mice. J Pain (2011) 12:370–83. 10.1016/j.jpain.2010.08.003 20889388

[58] Day YJ Liou JT Lee CM Lin YC Mao CC Chou AH Lack of interleukin-17 leads to a modulated micro-environment and amelioration of mechanical hypersensitivity after peripheral nerve injury in mice. Pain (2014) 155:1293–302. 10.1016/j.pain.2014.04.004 24721689

[59] Zhu W Acosta C MacNeil B Cortes C Intrater H Gong Y Elevated expression of fractalkine (CX3CL1) and fractalkine receptor (CX3CR1) in the dorsal root ganglia and spinal cord in experimental autoimmune encephalomyelitis: implications in multiple sclerosis-induced neuropathic pain. Biomed Res Int (2013) 2013:480702. 10.1155/2013/480702 24175290 PMC3794538

[60] Melanson M Miao P Eisenstat D Gong Y Gu X Au K Experimental autoimmune encephalomyelitis-induced upregulation of tumor necrosis factor-alpha in the dorsal root ganglia. Mult Scler (2009) 15:1135–45. 10.1177/1352458509106856 19667008

[61] Begum F Zhu W Cortes C MacNeil B Namaka M Elevation of tumor necrosis factor alpha in dorsal root ganglia and spinal cord is associated with neuroimmune modulation of pain in an animal model of multiple sclerosis. J Neuroimmune Pharmacol (2013) 8:677–90. 10.1007/s11481-013-9449-5 23483352

[62] Rodrigues DH Leles BP Costa VV Miranda AS Cisalpino D Gomes DA IL-1beta is involved with the generation of pain in experimental autoimmune encephalomyelitis. Mol Neurobiol (2015). 10.1007/s12035-015-9552-0 26614512

[63] Hao J Liu R Piao W Zhou Q Vollmer TL Campagnolo DI Central nervous system (CNS)-resident natural killer cells suppress Th17 responses and CNS autoimmune pathology. J Exp Med (2010) 207:1907–21. 10.1084/jem.20092749 20696699 PMC2931174

[64] Christy AL Walker ME Hessner MJ Brown MA. Mast cell activation and neutrophil recruitment promotes early and robust inflammation in the meninges in EAE. J Autoimmun (2013) 42:50–61. 10.1016/j.jaut.2012.11.003 23267561

[65] Gao M Yang Y Li D Ming B Chen H Sun Y CD27 natural killer cell subsets play different roles during the pre-onset stage of experimental autoimmune encephalomyelitis. Innate Immun (2016) 22:395–404. 10.1177/1753425916658111 27368310

[66] Zuo Y Perkins NM Tracey DJ Geczy CL. Inflammation and hyperalgesia induced by nerve injury in the rat: a key role of mast cells. Pain (2003) 105:467–79. 10.1016/S0304-3959(03)00261-6 14527707

[67] Sakamoto A Andoh T Kuraishi Y. Involvement of mast cells and proteinase-activated receptor 2 in oxaliplatin-induced mechanical allodynia in mice. Pharmacol Res (2016) 105:84–92. 10.1016/j.phrs.2016.01.008 26804251

[68] Nascimento D Pozza DH Castro-Lopes JM Neto FL. Neuronal injury marker ATF-3 is induced in primary afferent neurons of monoarthritic rats. Neurosignals (2011) 19:210–21. 10.1159/00033019533 21912089

[69] Braz JM Basbaum AI. Differential ATF3 expression in dorsal root ganglion neurons reveals the profile of primary afferents engaged by diverse noxious chemical stimuli. Pain (2010) 150:290–301. 10.1016/j.pain.2010.05.005 20605331 PMC2922479

[70] Ossipov MH Zhang ET Carvajal C Gardell L Quirion R Dumont Y Selective mediation of nerve injury-induced tactile hypersensitivity by neuropeptide Y. J Neurosci (2002) 22:9858–67. 12427842 10.1523/JNEUROSCI.22-22-09858.2002 PMC6757820

[71] Peirs C Williams SP Zhao X Walsh CE Gedeon JY Cagle NE Dorsal horn circuits for persistent mechanical pain. Neuron (2015) 87:797–812. 10.1016/j.neuron.2015.07.029 26291162 PMC4562334

[72] Xu ZZ Kim YH Bang S Zhang Y Berta T Wang F Inhibition of mechanical allodynia in neuropathic pain by TLR5-mediated A-fiber blockade. Nat Med (2015) 21:1326–31. 10.1038/nm.3978 26479925 PMC4752254

[73] Olechowski CJ Parmar A Miller B Stephan J Tenorio G Tran K A diminished response to formalin stimulation reveals a role for the glutamate transporters in the altered pain sensitivity of mice with experimental autoimmune encephalomyelitis (EAE). Pain (2010) 149:565–72. 10.1016/j.pain.2010.03.037 20399559

